# Hepatic Colic

**Published:** 1880-02

**Authors:** 


Hepatic Colic.—M. Laborde sums up as follows the results to which he has been led by his experimental physiological investigations as applied to therapeutics in regard to the subject of hepatic colic:

1. The excretory biliary ducts are endowed with contractility, and may therefore enter a spasmodic condition under the influence of excitations, direct or indirect; this contractility is of the nature of that of the smooth muscular fibres of organic life, and the existence of these fibres in the walls of the said ducts is demonstrated by histological anatomy, here perfectly in accord with experimental physiology.

2. The mucous membranes of these ducts are endowed with a very acute sensibility, revealing itself at the time under the influence of more or less intense excitations, by painful impression and expression, and by reflex phenomena of which the immediate manifestation is spasm of the canals.

3. These phenomena are especially brought about by the presence and contact of foreign bodies (biliary calculi,) and the spotaneous passage of these is from this same cause rendered very difficult, and only takes place, when it occurs, after a more or less long period of time, with the peculiarity that these bodies may pass back again into the gall bladder.

4. The so-called anæsthetic and antispasmodic medicines are most appropriate for the treatment of this morbid state, the conditions of which are easily produced experimentally.

5. These remedies, notably among them morphine, chloroform, and hydrate of chloral, act by exerting at once an anæsthetic and paralysant influence, whence results cessation of the spasm, the dilatation of the ducts and the accumulation of the biliary liquid, which acts on the foreign body as a *vis a tergo* propelling it toward the intestine.

6. The association of chlorate of morphine with chloroform or hydrate of chloral, that is, the simultaneous administration of these medicinal agents, forms the most efficient means of obtaining the results sought for, viz., the anæsthesia of the biliary ducts, preventing the pain and exerting a favorable influence for the migration and rapid passage of the foreign bodies.—(*Trib. Med*) *Gax.-des Hopitaux.*

